# Toughening Ionic Polymer Using Bulky Alkylammonium Counterions and Comb Architecture

**DOI:** 10.1021/acsmacrolett.2c00737

**Published:** 2023-03-24

**Authors:** Daisuke Aoki, Kento Yasuda, Koji Arimitsu

**Affiliations:** Department of Pure and Applied Chemistry, Tokyo University of Science, 2641 Yamazaki, Noda, Chiba 278-8510, Japan

## Abstract

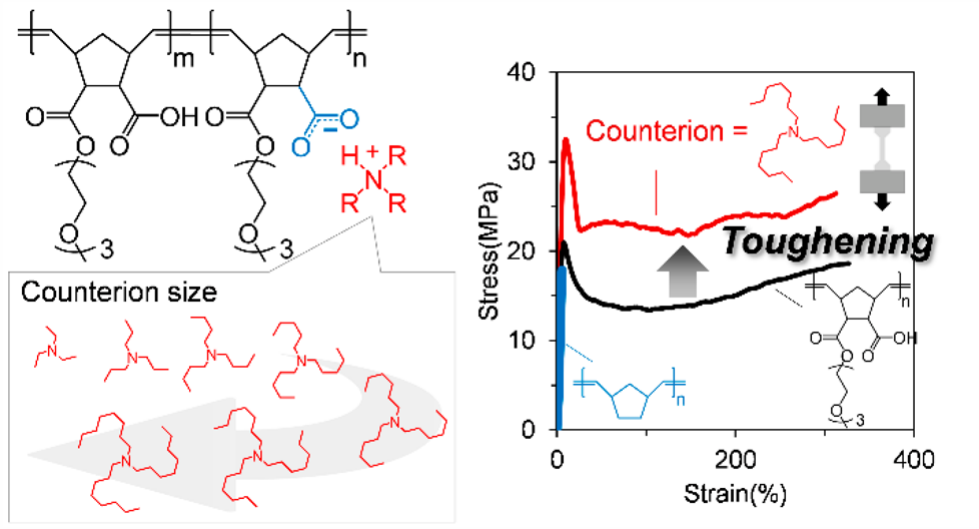

Ionic interactions in ionic polymers, such as ionomers, polyelectrolytes, and polyampholytes, contribute to toughness in systems with high mobility and active ion dynamics, such as hydrogels and elastomers. However, it remains challenging to toughen rigid polymers through ionic interactions without lowering their elastic modulus through plasticization. Here, we present a strategy for toughening without sacrificing the elastic modulus by combining a comb polymer with bulky ammonium counterions. We designed and synthesized ionic comb polymers with oligoethylene glycol side chains and carboxylic acids in each monomer unit of the polynorbornene backbone, neutralized by trialkylamines, ranging from ethyl to octyl. The counterion size in ionic comb polymers influenced the mechanical properties of tensile testing—not the elongation at break and the elastic modulus but the ultimate strength and toughness. The ionic comb polymer containing heptylammonium counterions displayed the highest toughness of 77 MJ m^–3^. Tensile studies at various strain rates demonstrated a rate-dependent difference between heptyl- and octylammonium counterions. This result suggests that the heptylammonium counterion acted as a sacrificial bond by providing a moderate dissociation rate that was slightly slower than that of the octylammonium counterion, leading to toughening.

Cross-linked polymers have been widely used in industry due to their excellent mechanical properties and chemical stability. However, it remains challenging to achieve both high modulus and hardness. Sacrificial bonds, reported by Gong et al., emerged as an excellent concept for the design of toughening cross-linked hydrogels because they can dissipate fracture energy by preferentially breaking as weak bonds.^1,2^ This idea has been extended in various weak bonds, such as ionic interactions,^3^ hydrogen bonding,^4,5^ metal coordination bonds,^6^ and dynamic covalent bonds,^7^ and is applicable to dry networks as well as to hydrogels. Among them, ionic interactions differentiate from other dissociative interactions in that the bulk properties of polymers are governed by the dynamics of the ionic network.^8^ These dynamics are dependent on a variety of parameters including the quantity, nature, strength, and distance of ionic motifs as well as the chemical structure of the polymer backbone.^9−11^ Notably, ionic soft polymers with low glass transition temperatures (*T*_g_) allow for active ion dynamics, resulting in extraordinarily tough soft materials.^12,13^ However, due to the limited mobility of polymers in rigid polymer networks with a high *T*_g_, it is difficult to develop a universal strategy for toughening using ionic interactions without a decrease in elastic modulus.

To address the invalidation of ionic interactions in rigid polymer systems, we present an ionic toughening strategy that facilitates ion exchange by combining bulky alkylammonium counterions with comb polymers composed of excess carboxylic acids and polar soft side chains. Because their alkyl chains partially mask electrostatic interactions, bulky alkylammonium counterions behave like plasticizers, preventing the formation of typical ionic aggregate structures^14^ and thereby lowering *T*_g_, reducing melt viscosity, and softening mechanical properties.^15,16^ Moreover, excess carboxylic acids and polar soft side chains also assist ion exchange by accelerating the rate of ion hopping^17,18^ and preventing excessive ionic aggregation.^16^

In this study, we synthesized comb polymers with carboxylic acid consisting of a polynorbornene backbone and oligo ethylene glycol (OEG) side chains and prepared ionic comb polymers by combining trialkylamines as counterions with different alkyl chain lengths. Surprisingly, trialkylammonium counterions with appropriate bulkiness toughened ionic comb polymers without compromising the elastic modulus or elongation at break.

To study the mechanical properties of ionic polymers with ionic groups and side chains, we first designed and synthesized ionic polynorbornene with OEG side chains and carboxylic acids in each monomer unit (Scheme 1). Due to the ease of ring-opening metathesis polymerization by Grubbs catalyst and the inertness of olefins to various chemical groups, the polynorbornene backbone has been the preferred choice for many reported graft polymers. For the OEG side length, we chose a methyl-terminated trimeric OEG (OEG_3_) to provide typical plastic-like mechanical behavior to comb polymers containing carboxylic acids prior to neutralization (Figure S10). We synthesized ionic comb polymers by grafting OEG_3_ onto polynorbornene-containing dicarboxylic anhydride to modify each monomer with OEG_3_ side chains and carboxylic acids in equal quantities. Although norbornene dicarboxylic anhydride (NBC) is a low-cost and rational starting material for our synthetic strategy, there are few reports for homopolymerization due to the low reactivity, the poor solubility, and an extremely high glass transition temperature of its polymer.^19,20^ We successfully produced poly NBC (pNBC) by reacting a high concentration of NBC with a third-generation Grubbs catalyst at room temperature in DMF. ^1^H NMR spectra demonstrated the polymerization of NBC by chemical shifts from ring-closed olefins of around 6.3 ppm to ring-opened olefins of around 5.3 and 5.5 ppm, as well as a broadening of the overall peak (Figures 1a and b). The subsequent esterification reaction of pNBC with mono alcohol OEG_3_ catalyzed by DMAP was almost 100% efficient, as evidenced by the formation of a new methylene peak adjacent to the ester at around 4.0 ppm in the ^1^H NMR spectrum (Figure 1c). The progress of the pNBC esterification reaction is also confirmed by the presence of COOH in the FTIR spectrum and the shift of the C=O stretching vibration peak to lower wavenumbers (Figure S2). In addition, the resultant comb polymer containing carboxylic acid (pNBC-*g*) had an average molecular weight of 1.22 × 10^5^ Da estimated by the polystyrene equivalent with THF SEC (Figure S1). Finally, the ionic comb polymers were produced by neutralizing a pNBC-*g* solution with trialkylamines ranging in size from Et_3_N to Oc_3_N. Figure 1d illustrates a typical ^1^H NMR spectrum for pNBC-*g*-Et_3_N. Even after purification of the pNBC-*g*-Et_3_N, the presence of neutralized trialkylamine peaks provides evidence for the neutralization of carboxylic acids by Et_3_N, which is further confirmed by the presence of a carboxylate peak at 1564 cm^–1^ in the FTIR spectrum (Figure S2). All pNBC-*g*-base samples were estimated to be 20–30% neutralized by H NMR and were kept in desiccators to minimize moisture absorption. Moreover, the *T*_g_ of pNBC-*g* was determined to be 87 °C using temperature-dependent rheological measurements, although differential scanning calorimetry was unable to identify it (Figure S18). In contrast, the *T*_g_ of the pNBC-*g*-base declined to 68 °C with increasing counterion size, indicating plasticization, similar to previous studies involving alkylammonium counterions.

**Scheme 1:**
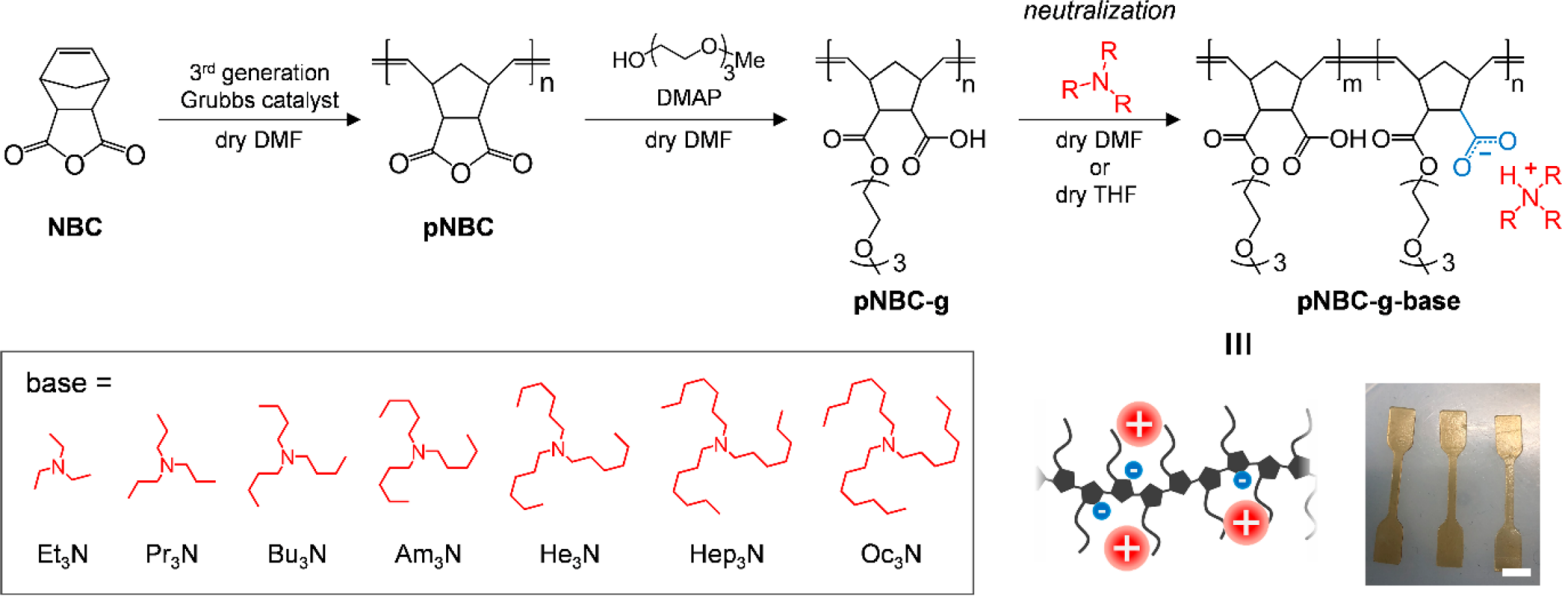
Synthesis of Ionic Comb Polymers (pNBC-g-base) with Various Sizes of Alkylammonium Counterions from Et_3_N to Oc_3_N White scale bar indicates 6 mm.

**Figure 1:**
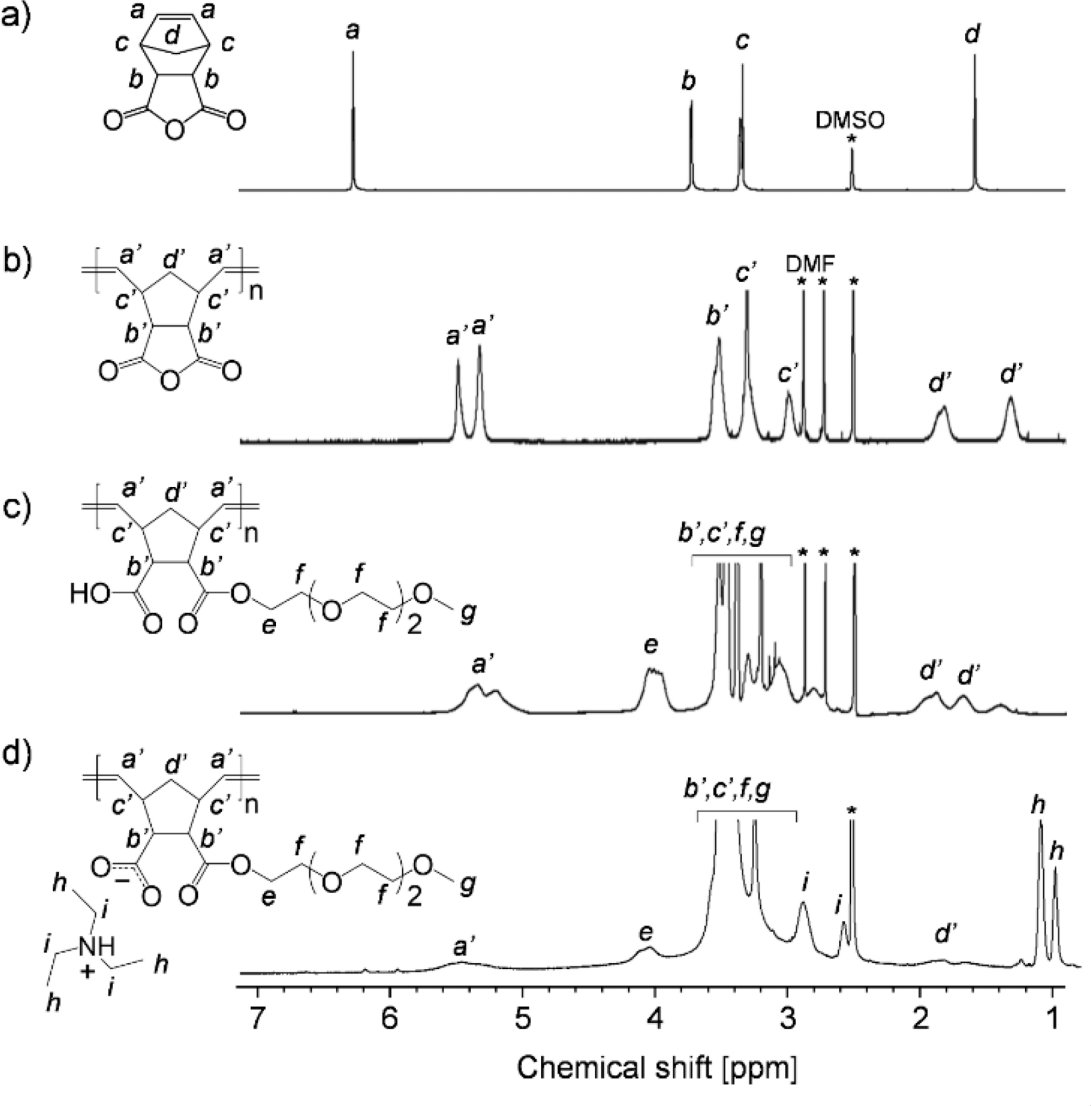
^1^H NMR spectra for (a) NBC, (b) pNBC, (c) pNBC-*g*, and (d) pNBC-*g*-Et_3_N in DMSO-*d*_6_ (400 MHz).

Next, we move to investigate the tensile mechanical properties of the pNBC-*g* base samples neutralized by varying ammonium counterion sizes. Figure 2a depicts the stress–strain curves of the pNBC-*g* base films with trialkylammonium of various alkyl chain lengths from ethyl to octyl. Interestingly, even though larger ammonium counterions generally plasticize the polymer,^21^ the elongation at break was almost the same independent of counterion sizes, with only the stress differing. Figure 2b summarizes the ultimate strength (σ_b_) and elongation at break (ε_b_) as a function of trialkylammonium counterion sizes. Depending on the counterion size, the neutralization of carboxylic-acid-containing comb polymers by alkylamines decreased, remained constant, or increased the σ_b_. For pNBC-*g*-Oc_3_N, low stress level deformation appears with a small yielding stress (σ_y_) of about 11–13 MPa and eventually breaks at about 10–15 MPa. pNBC-*g* base with moderate counterion sizes in the range of Et_3_N to Hex_3_N had almost the same σ_y_ and σ_b_ of 15–25 MPa as comb norbornene without ion neutralization. Remarkably, pNBC-*g*-Hep_3_N deformed while maintaining a high level of stress, demonstrating σ_y_ and σ_b_ of 25–30 MPa. This mechanical strengthening effect of the heptylammonium counterion while maintaining its ductile properties also resulted in an increase in toughness. Figure 2c shows the toughness (*U*_t_) calculated from the area of the stress–strain curve as a function of counterion sizes. Toughness follows a nonlinear trend, with pNBC-*g*-Hep_3_N exhibiting the highest *U*_t_ (77 MJ m^–3^), comparable with commercial polycarbonate and previously reported toughened polynorbornene derivatives^5^ (Figure 2c). Incidentally, the neutralization ratio had a significant effect on the mechanical properties; when the Hep_3_N fraction exceeded 40%, the toughness decreased dramatically (Figure S14). In addition, the elastic modulus of a series of pNBC-*g* bases was comparable with that of carboxylic comb norbornene, except for pNBC-*g*-Oc_3_N (Figure 2d). Thus, the appropriate ion size enables the toughening of ionic comb polymers by increasing only the stress level, without reducing the elastic modulus and ductility. Moreover, to understand the contribution of OEG side chains to toughening, we synthesized a model polymer without side chains, called Me, in which the OEG3 side chain was substituted by a methyl ester. Figure 2e shows the stress–strain curves of pNBC-*g*, pNBC-*g*-Hep_3_N, polynorbornene (pNB), and Me. pNBC-*g*-Hep_3_N combines high σ_b_ and ε_b_ in comparison to pNB, pNBC-*g*, and Me, improving the *U*_t_ of pNB without side chains and counterions by about 192 times. The toughened pNBC-*g*-Hep_3_N displayed transparent deformation up to 200% elongation beyond yielding in the plateau region of the stress–strain curve, followed by whitening, indicating the production of crazes during strain hardening (Figure 2f). Additionally, using Me-Hex_3_N (Me neutralized with Hex_3_N), we also examined whether the combination of a bulky counterion and a side chain is essential for toughening. Figure 2g represents the stress–strain curves of Me, Me-Hex_3_N, pNBC-*g*, and pNBC-*g*-Hex_3_N. Despite the similar tensile behavior of pNBC-*g* and Me in the absence of counterions, the presence of counterions had opposing effects on mechanical properties. Despite possessing counterions with long alkyl chains, Me-Hex_3_N exhibited brittle mechanical behavior, whereas pNBC-*g*-Hex_3_N exhibited tensile behavior similar to pNBC-*g*. The *U*_t_ calculated from these stress–strain curves is compared in Figure 2h. Despite pNBC-*g*-Hex_3_N displaying a similar *U*_t_ value to pNBC-*g*, the neutralization of Hex_3_N to Me without side chains decreased *U*_t_ from 37 to 1 MJ m^–3^. These results suggest that the presence of side chains promotes the dissociation of bulky alkylammonium from the rigid norbornene backbone, allowing for the formation of sacrificial bonds.

**Figure 2:**
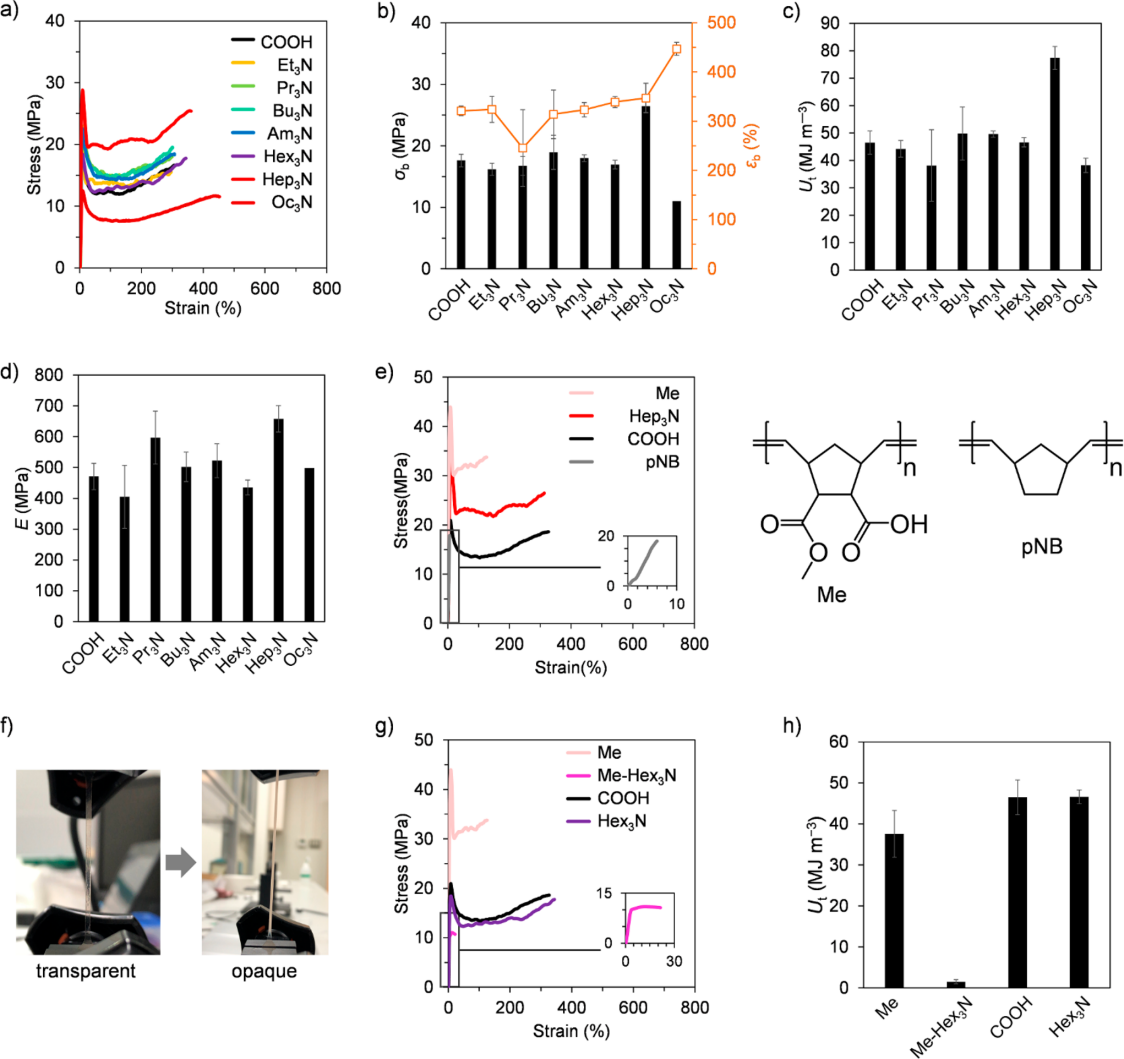
Stress–strain curves of pNBC-*g* base compared with (a) counterion sizes, (e) side chains and carboxylic acids, and (g) side chains and counterions. Comparison of mechanical properties obtained from the tensile curves of pNBC-*g* base in terms of (b) ultimate strength (σ_b_) and elongation at break (ε_b_), (c) toughness (*U*_t_), and (d) elastic modules (*E*). (f) Optical images showing the tensile test of pNBC-*g*-Hep_3_N. (h) Comparison of toughness of Me, Me-Hex_3_N, pNBC-*g*, and pNBC-*g*-Hex_3_N. The names of the alkylamines and COOH indicate the counterions and pNBC-*g*.

To comprehend the highest strength and toughness of the heptyl counterion, we studied ion exchange rates as estimated by tensile testing at different crosshead speeds and rheological studies. According to Leibler et al., the mechanical behavior of dynamically cross-linked polymers is strain rate dependent because dynamic cross-links behave as cross-linking points on time scales shorter than their lifetime and become viscoelastic on longer time scales.^22^Figure 3a displays plots of normalized yielding strength based on 1 mm/min as a function of strain rate for pNBC-*g* base with various counterions. A higher normalized yield strength indicates a larger increase in yield strength when deformed at a faster rate than 1 mm/min. Based on Leibler’s theory,^22^ the higher the normalized yield strength, the faster the exchange rate of reversible bonds, which in this study means a faster ion exchange rate. Comparing triethylamine, trihexylamine, and heptylamine, the strain rate dependency of the normalized yield strength showed little difference within the measured range except for the initial slope magnitude. In contrast, from heptyl to octyl, the normalized yield strength became significantly more sensitive to strain rate and increased dramatically with exponential decay. This tendency indicates that the ion exchange rate of octyl is significantly more active than that of other alkylamines shorter than heptyl, which is supported by the softer stress–strain behavior of pNBC-*g*-Oc_3_N (Figure 2a). Interestingly, our pNBC-*g*-Hep_3_N, which has one less carbon atom than this softening pNBC-*g*-Oc_3_N, offers the best strength and toughness without compromising elastic modulus and elongation at break. Note that the effective cross-link density in relation to the mechanical properties should be nearly identical from propyl to heptyl, as the swelling ratio was almost the same (Figure S15). Even though the cross-link densities were almost the same, only pNBC-*g*-Hep_3_N grew tougher. This is most likely due to the cross-linking points dissociating at the appropriate rate, allowing them to behave as sacrificial bonds that break preferentially during deformation.

**Figure 3:**
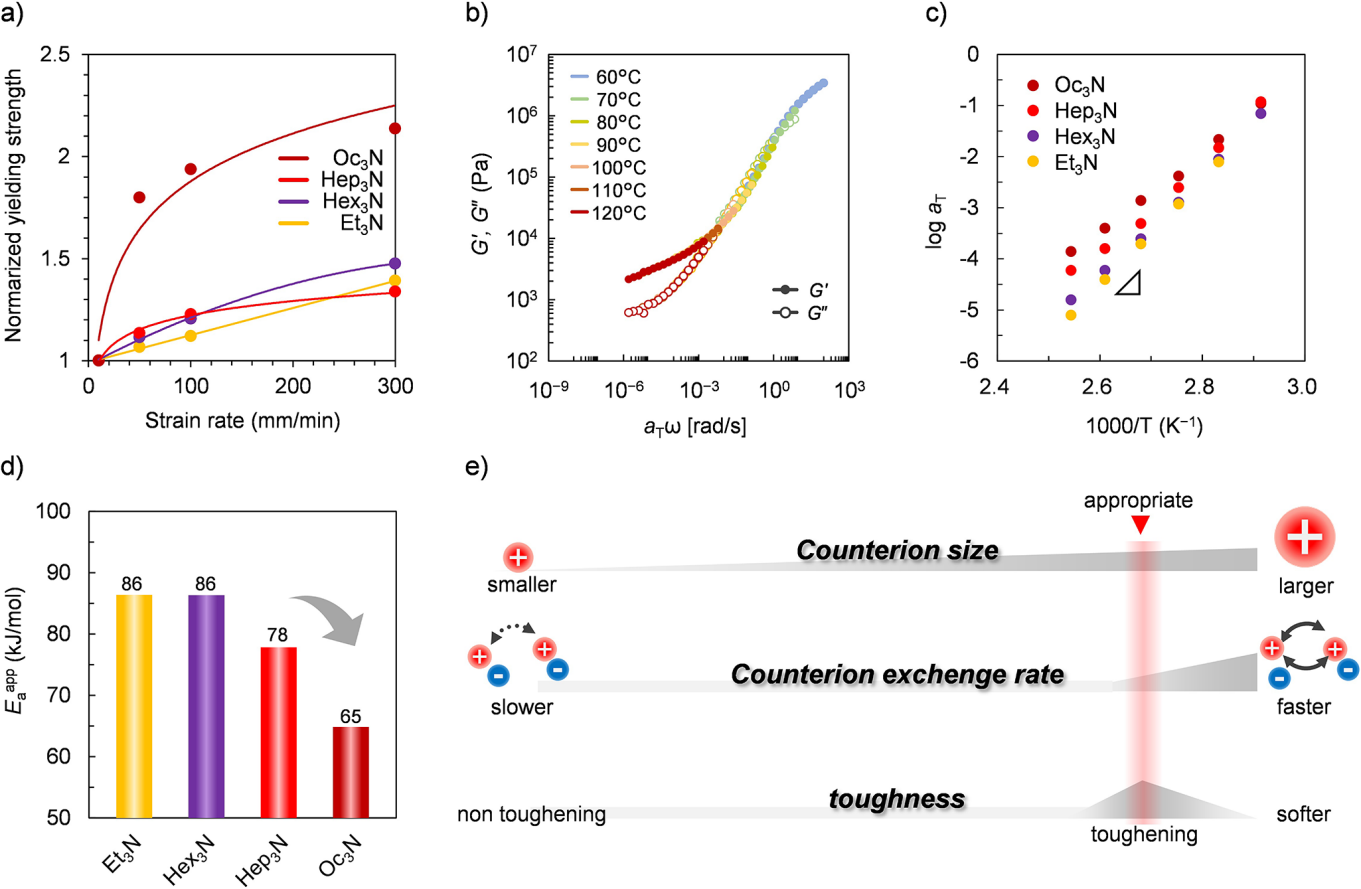
(a) Plots of normalized yielding strength based on 1 mm/min as a function of strain rate for pNBC-*g*-Et_3_N, pNBC-*g*-Hex_3_N, pNBC-*g*-Hep_3_N, and pNBC-*g*-Oc_3_N. (b) Representative frequency dependence of storage (*G*′) and loss modulus (*G*″) master curves at *T*_r_ = 60 °C for pNBC-*g*-Hex_3_N. (c) Arrhenius plots of shift factors (*a*_T_) for pNBC-*g* base samples at *T*_r_ = 60 °C. (d) Apparent activation energies (*E*_a_^app^) determined by Arrhenius plots. (e) Schematic illustration of our possible mechanism: the larger counterions are the faster ion exchanges.

Moreover, we evaluated the variation in ion exchange rate with counterion size by analyzing the frequency-dependent dynamics in rheological measurements. Miwa et al. explained the softening of mechanical behavior at high ion exchange rates estimated by relaxation times of network rearrangement using polymers, whose ion exchange rate varies depending on a gas atmosphere.^23^Figure 3b exhibits a representative master curve of the storage and loss modulus for pNBC-*g*-Hex_3_N at *T*_r_ = 60 °C. Time–temperature superposition (tTs) was successfully applied to all samples, including pNBC-*g*-Et_3_N, pNBC-*g*-Hex_3_N, pNBC-*g*-Hep_3_N, and pNBC-*g*-Oc_3_N, within the temperature range of 60–120 °C (Figure S17). The reversible gelation model by Chen et al.^24^ in the terminal flow with *G*′ ∝ ω^2^ and *G*′′ ∝ ω can be used to determine the ionic dissociation time in the rheological studies. Unfortunately, even at high temperatures up to 200 °C, the pNBC-*g* base samples remained in the rubber region (*G*′ > *G*″), presumably due to the long termination relaxation times caused by the high concentration of COOH content in each pNBC monomer. Notably, the regions where *G*′ and *G*″ values were almost the same indicate the presence of an equilibrium of trapped strands, which is a characteristic of supramolecular polymers with typical sticky side groups.^25^ Because the pNBC-*g* base samples behave as supramolecular polymers, we attempted to calculate the apparent activation energy (*E*_a_^app^) of reversible gels with strong association using the Rubinstein–Semenov theory^26^ in order to assess the ion exchange rate. The *E*_a_^app^ was extracted by Arrhenius plots of the shift factor at 60–120 °C when the tTs is valid (Figure 3c).^27^ The resultant *E*_a_^app^ was nearly the same for Et_3_N and Hex_3_N, at 86 kJ/mol, but dramatically decreased for Hep_3_N and Oc_3_N, at 78 and 65 kJ/mol, respectively (Figure 3d). The consistent *E*_a_^app^ values for Et_3_N and Hex_3_N support the nearly identical stress–strain curve trends of pNBC-*g*-Et_3_N and pNBC-*g*-Hex_3_N shown in Figure 2a. Because larger counterions have faster molecular dynamics,^14^ there probably exists a threshold between counterion sizes of Hex_3_N and Hep_3_N like a critical chain length effect, at which counterion exchange becomes active. Oc_3_N exhibited significantly lower *E*_a_^app^ than Hep_3_N, indicating a faster dissociation rate compared to the other shorter counterion. The active exchange of counterions in pNBC-*g*-Oc_3_N is primarily responsible for its softness, leading to low stress levels and a strong dependence on strain rate in tensile testing. The significant contrast in mechanical properties between Hep_3_N and Oc_3_N counterions necessitates an appropriate counterion exchange rate for effective toughening through ionic interactions in a stiff polymer with a high *T*_g_ (Figure 3e). Such an exchange rate can facilitate sacrificial bonding in the material, dissipating fracture energy and thereby enhancing toughness. Therefore, even rigid polynorbornene backbones can be efficiently toughened by combining bulky counterions and comb architecture to enhance ion dissociation rates.

In conclusion, combining bulky alkylammonium counterions with a comb architecture has enabled the successful toughening of a rigid polynorbornene derivative while maintaining their high elastic modulus, based on the notion that high counterion mobility promotes toughness even in high *T*_g_ polymers. We designed and synthesized pNBC-*g* base, an ionic comb polymer incorporating alkylammonium counterions with alkyl chain lengths ranging from ethyl to octyl. The neutralization ratios of these synthesized pNBC-*g* bases were determined to be 20–30% using ^1^H NMR. In the stress–strain curves for the pNBC-*g* bases, the elongation at break and elastic modulus were nearly constant regardless of ion size; however, the overall stress level and toughness changed with ion size. Remarkably, pNBC-*g*-Hep_3_N displayed a toughness of 77 MJ m^–3^, which is 1.7 times that of pNBC-*g* and 192 times that of a typical norbornene pNB. To explain this toughening, the rate dependence of stress associated with the dissociation rate of dynamic bonds was studied. Octylammonium counterions displayed a greater dependence on rate than other counterions, indicating a fast dissociation. The heptylamine, which is only one carbon shorter than octylamine, was toughened because it works as a sacrificial bond because its dissociation rate was not extremely rapid like octyl’s, but rather appropriate. This research has paved the way for toughening even rigid polymers without compromising their elastic modulus by promoting ionic interaction dissociation rates. If the critical factors for toughness can be quantified in conjunction with rheology and X-ray spectroscopy, this work will contribute to a more universal strategy for toughness for a variety of rigid polymer network materials, not just bulky counterion and comb architectural combinations.

## Supporting Information Available

Materials and instruments; experimental procedures; polymerization procedures; SEC profile; FTIR spectra; stress–strain curves; and swelling study (PDF)
