# Supplementary material for: Toughening Ionic Polymer Using Bulky Alkylammonium Counterions and Comb Architecture
Source: ACS Macro Lett. 2023 Mar 24;12(4):462–7. doi: 10.1021/acsmacrolett.2c00737 (PMC10116644; doi:10.1021/acsmacrolett.2c00737)
Supplement: Supplementary file 1 — mz2c00737_si_001.pdf [file mz2c00737_si_001.pdf]

Supporting information

Toughening ionic polymer using bulky alkylammonium counterions and comb architecture

*Daisuke Aoki, Kento Yasuda, Koji Arimitsu**

Department of Pure and Applied Chemistry, Tokyo University of Science, 2641 Yamazaki, Noda, Chiba 278-8510, Japan.

*email: arimitsu@rs.tus.ac.jp

1. Materials and instruments.

1.1 Materials

5-Norbornene-2,3-dicarboxylic anhydride (NBC), triethylene glycol monomethyl ether ($mOEG_3$), polyethylene glycol monomethyl ether 400, polyethylene glycol monomethyl ether 1000, 4-dimethylaminopyridine (DMAP), ethyl vinyl ether, triethylamine (Et_3N), tripropylamine (Pr_3N), tributylamine(Bu_3N), triamylamine (Am3N), trihexylamine (Hex3N), triheptykamine (Hep_3N), and trioctylamine (Oc_3N) were purchased from Tokyo Chemical Industry Co., Ltd. Dichloro[1,3-bis(2,4,6-trimethylphenyl)-2-imidazolidinylidene](benzylidene)bis(3-bromopyridine)ruthenium(II) (3rd generation Grubbs Catalyst, M300), ion exchange resin (Amberlyst FPC3500), and dimethyl sulfoxide-d_6 were purchased from Sigma Aldrich. *N,N*-Dimethylformamide, super dehydrated (DMF), tetrahydrofuran, super dehydrated, stabilizer free (THF), diethyl ether, and methanol were purchased from Wako Pure Chemical Industries.

1.2 Polymerization of pNBC

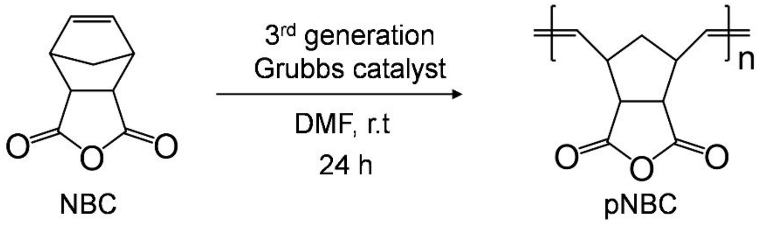

5-Norbornene-2,3-dicarboxylic anhydride (NBC) (40 g, 240 mmol) was placed in a 500-mL flask and the atmosphere was replaced with nitrogen. Then 110 mL of anhydrous DMF was added, and 10 mL of a DMF solution of Grubbs G3 (86 mg, 9.7×10^{-5} mmol) was added using a syringe. The mixture was then stirred at room temperature under a nitrogen atmosphere for 24 h. The reaction was quenched with 10 mL of ethyl vinyl ether, and the resulting polymer was purified by reprecipitation with an excess amount of THF and redissolution in DMF three times, and dried in vacuo at 100 °C.

1.3 Synthesis of pNBC-g

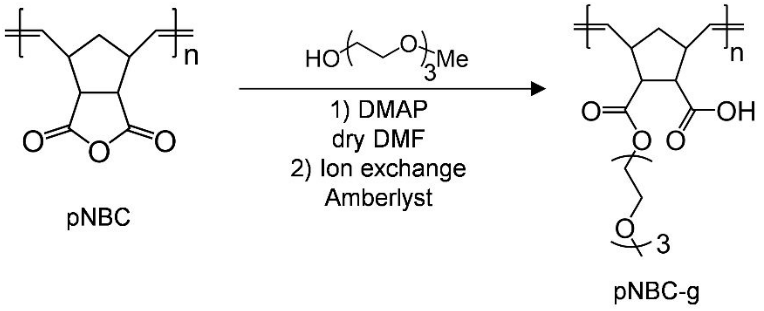

pNBC (12 g) was dissolved in 140 mL of anhydrous DMF, triethylene glycol monomethyl ether (mOEG$_3$) (24 g, 144 mmol) and 4-dimethylaminopyridine (DMAP) (6.4 g, 54 mmol) were added, and the mixture was stirred at 80°C for 10 h. The resulting polymer was reprecipitated with an excess amount of diethyl ether. Then, the precipitated polymer was dissolved in methanol, 40 mL of cation exchange resin was added and stirred at room temperature for 5 h to remove the DMAP counterions. This removing procedure was repeated twice. After evaporating the methanol, anhydrous THF or anhydrous DMF was added to the precipitated polymer and kept in solution. Note that vacuum drying will cause irreversible cross-linking and gelation.

1.4 Synthesis of pNBC-g-base

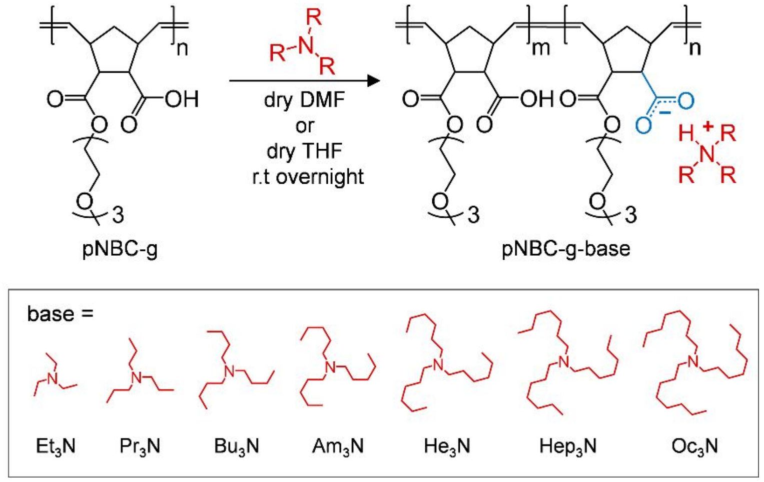

Alkylamines were added to the THF solution of PNBC-g-PEG3 in the case of Hep$_3$N and Oc$_3$N, and to the DMF solution in the other cases to achieve 20–30% neutralization ratio and stirred at room temperature overnight. The acid/base ratios for each trialkylamine neutralization are shown in Table S1. The resulting polymer was then purified by reprecipitation with an excess amount of diethyl ether.

Table S1. Summary of acid/base ratios and solvent used in each trialkylamine neutralization.

Base	Et_3N	Pr_3N	Bu_3N	Am_3N	Hex_3N	Hep_3N	Oc_3N
Acid / Base	2.70	0.27	0.27	0.36	0.40	0.72	0.72
Solvent	DMF	DMF	DMF	DMF	DMF	THF	THF

1.5 Measurements.

^{1}H NMR spectra were acquired in DMSO-d_6 using a JEOL ECZ-500. The Fourier transform infrared spectrometry (FT-IR) spectra were recorded using a JASCO FT-IR600. The number average molecular weight (M_n) and polydispersity index (M_w/M_n) were calculated using a gel permeation chromatography (GPC) with a L-2350 column oven, a L-2130 pump, and a L-2490 RI detector (HITACHI). The system was run using KF-805L narrow dispersed polystyrene standards (Shodex) and THF as the eluent at a temperature of 40°C. Tensile tests were carried out on dog bone test pieces cut from a punched blade JIS K6251 using an A&D Co., Ltd MCT-2150 with a crosshead speed of 10 mm/min. The swelling ratio (Q) of pNBC-g-bases in methanol was determined by an equation as $Q = (W_s\text{-}W_d)/W_d \times 100$, where W_s and W_d are the mass of swollen and dry pNBC-g-bases weighed for five samples after wiping off water using papers.

Round disc samples with a diameter of approximately 25 mm and thickness of approximately 1 mm were subjected to rheological measurements using a modular compact rheometer MCR302 (Anton Paar) equipped with a 25 mm cone plate (Figure S16). The samples were obtained by casting the polymer MeOH solution in PTFE petri dishes. Dynamic frequency sweeps were carried out with a frequency range of 0.1–100 rad/s and temperature range of 60−120 °C, using a constant strain of 0.1%. Master curves for all samples were constructed using time-temperature superposition (tTs) at a reference temperature of T_r = 60 °C.

2. Characterization

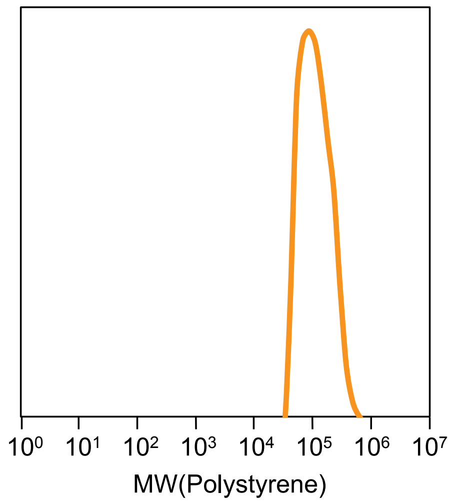

Figure S1. SEC profile for pNBC-g.

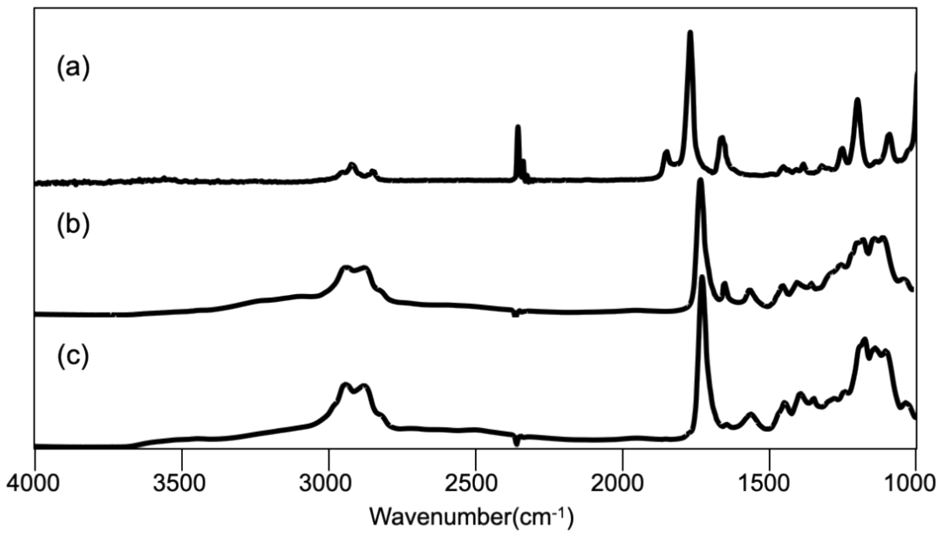

Figure S2. FT-IR spectra for (a) pNBC, (b) pNBC-g, (c) pNBC-g-Et_3N.

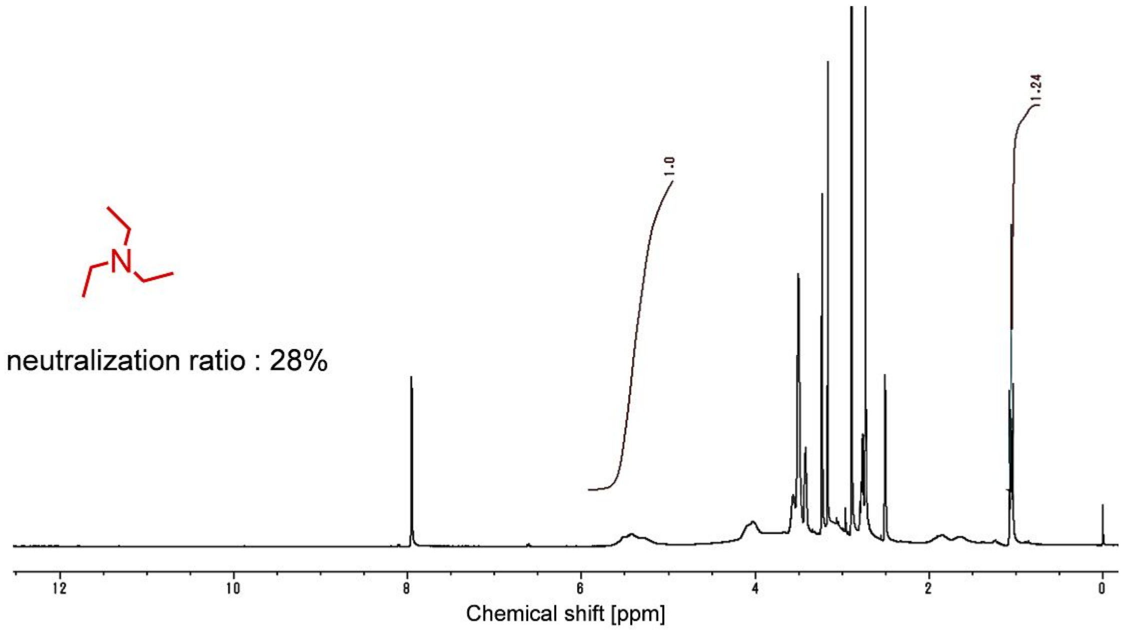

Figure S3. ^{1}H NMR spectrum for pNBC-g-Et_3N in DMSO-d_6 (500 MHz).

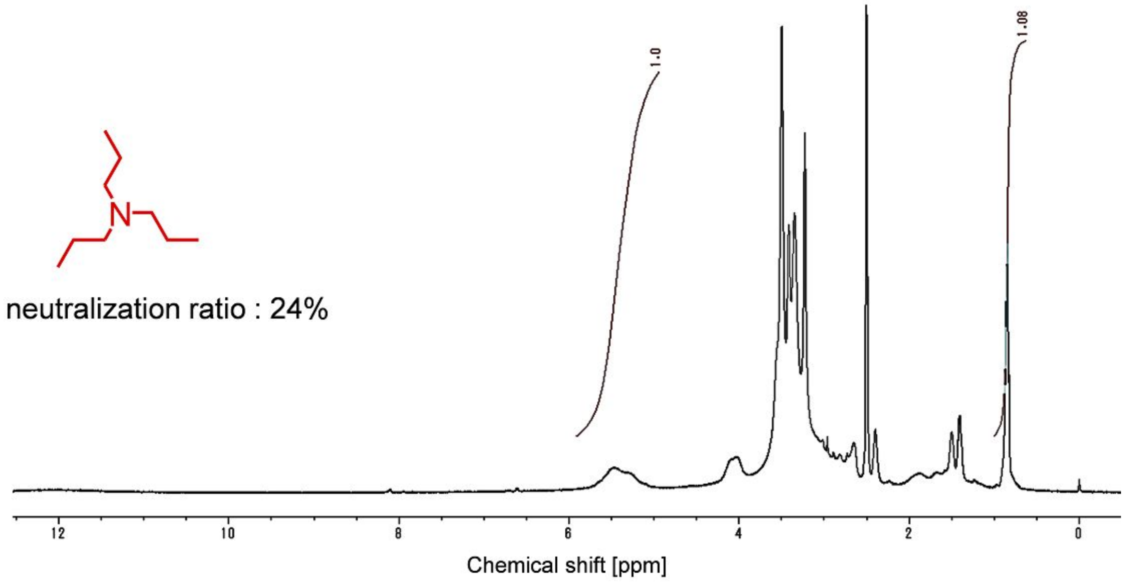

Figure S4. ^{1}H NMR spectrum for pNBC-g-Pr_3N in DMSO-d_6 (500 MHz).

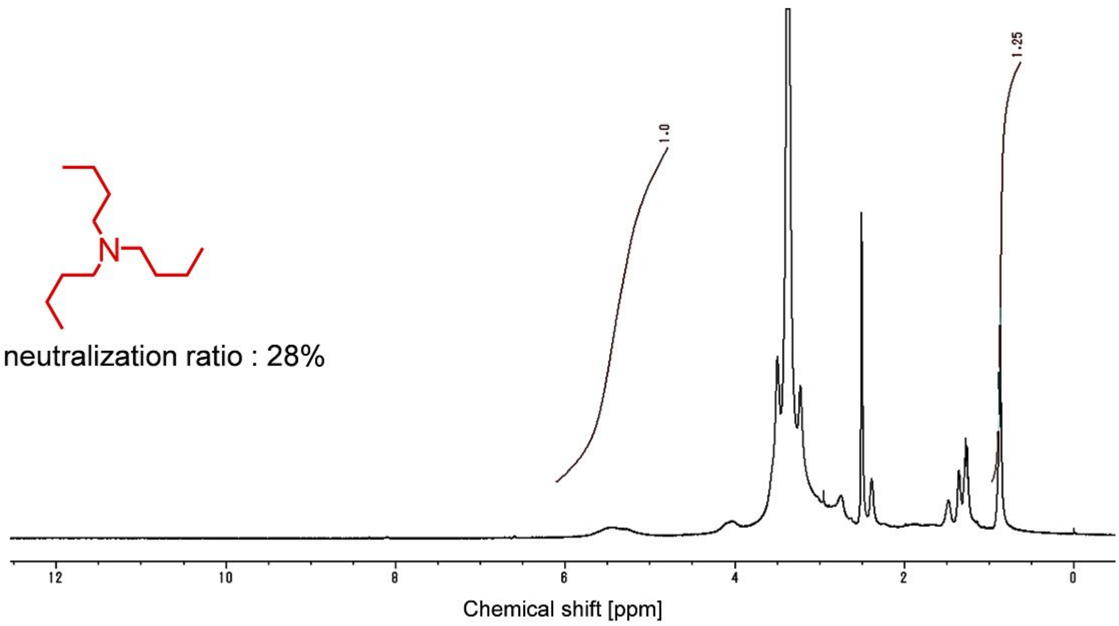

Figure S5. ^{1}H NMR spectrum for pNBC-g-Bu_3N in DMSO-d_6 (500 MHz).

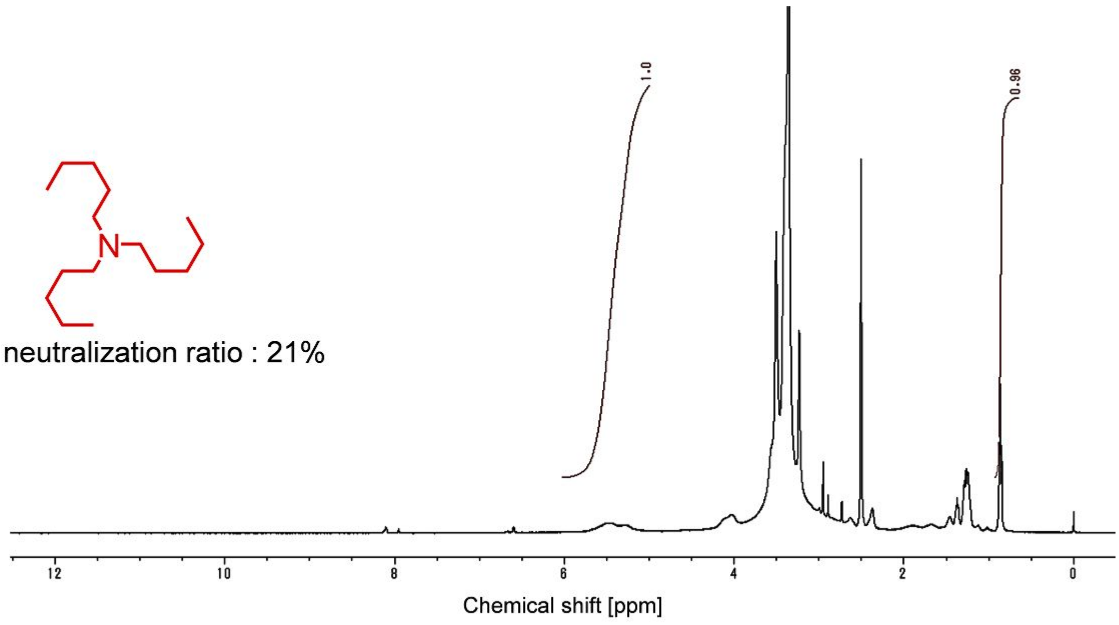

Figure S6. ^{1}H NMR spectrum for pNBC-g-Am_3N in DMSO-d_6 (500 MHz).

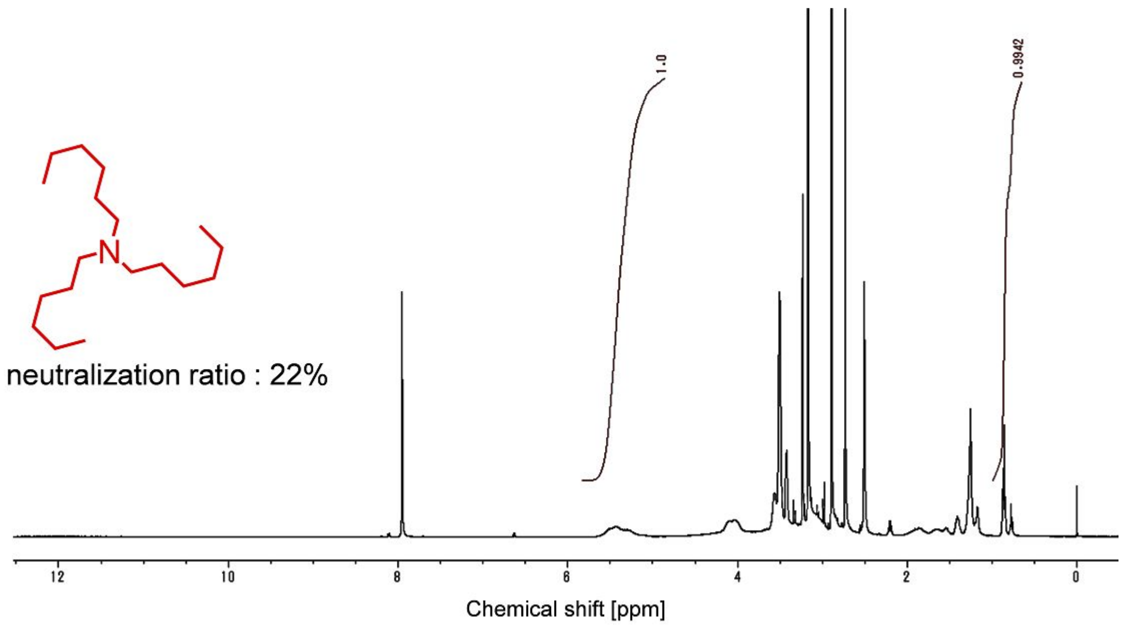

Figure S7. ^{1}H NMR spectrum for pNBC-g-Hex_3N in DMSO-d_6 (500 MHz).

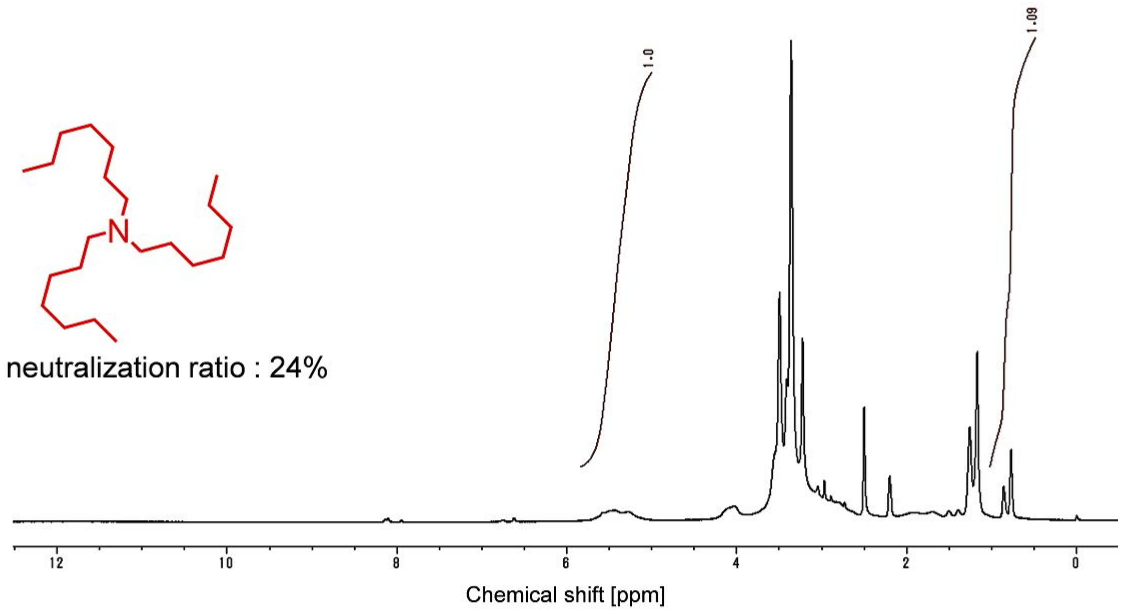

Figure S8. ^{1}H NMR spectrum for pNBC-g-Hep_3N in DMSO-d_6 (500 MHz).

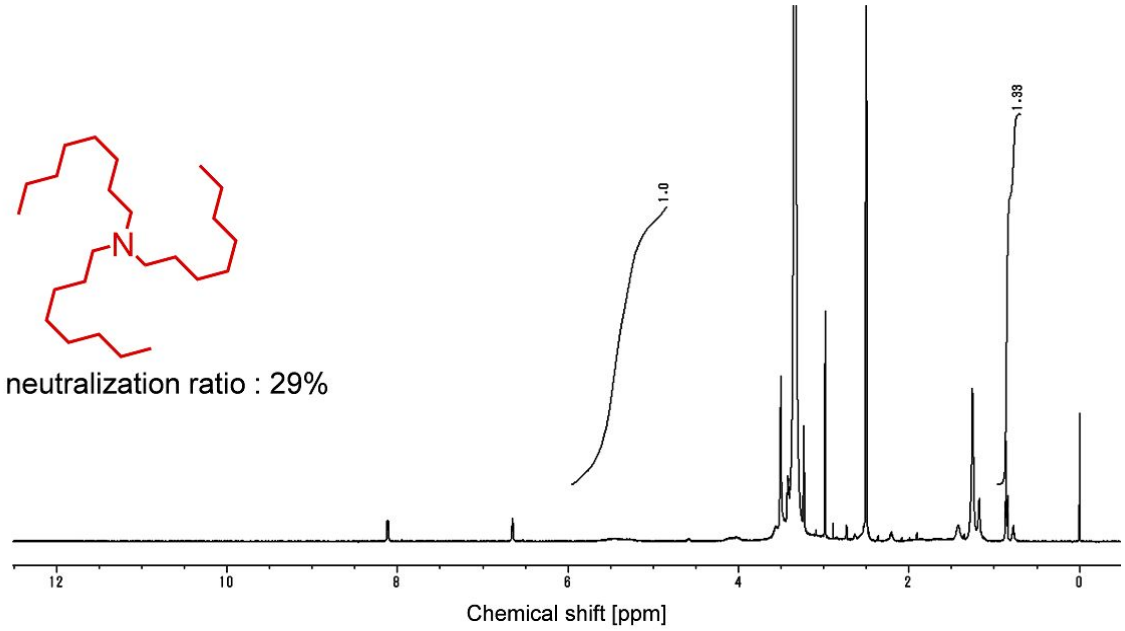

Figure S9. ^{1}H NMR spectrum for pNBC-g-Oc_3N in DMSO-d_6 (500 MHz).

3. Mechanical properties

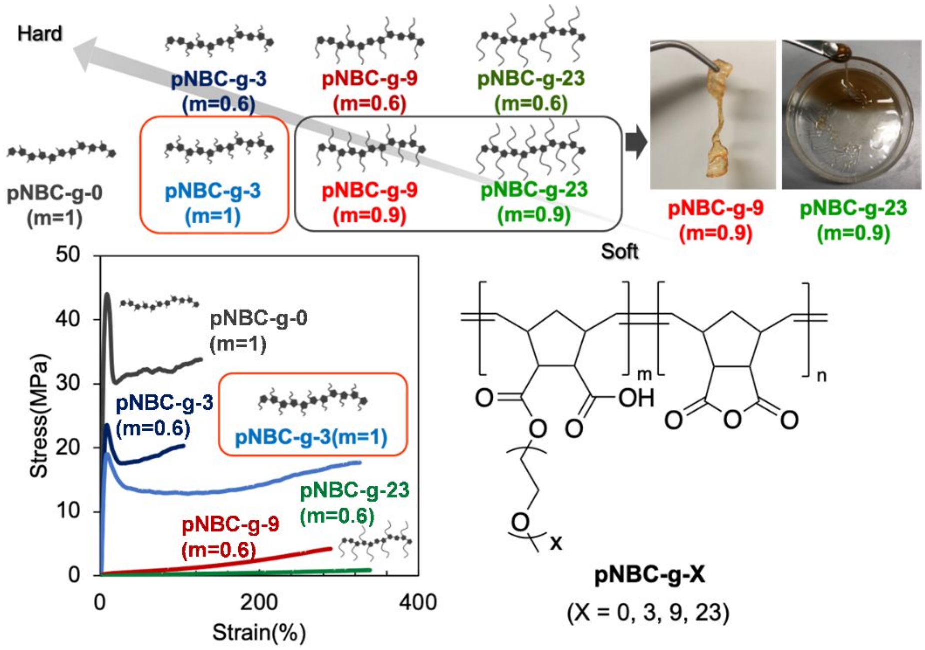

Figure S10. Stress-strain curves for comb polymers containing carboxylic acids with different side chain length and introduction ratio of side chains. A sample name pNBC-g-X indicates the average degree of polymerization of PEG in the side chain is “X”.

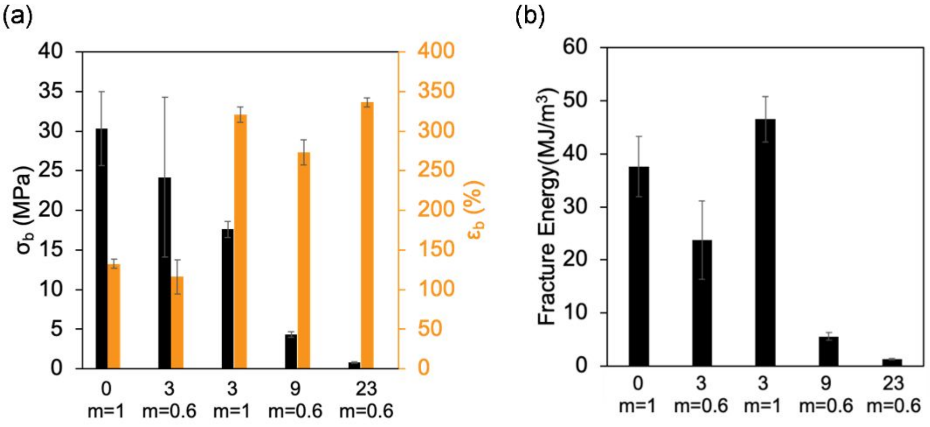

Figure S11. Comparison of mechanical properties obtained from the tensile curves of pNBC-g-X in terms of (a) ultimate strength (σ_b) and elongation at break (ε_b), and (b) fracture energy.

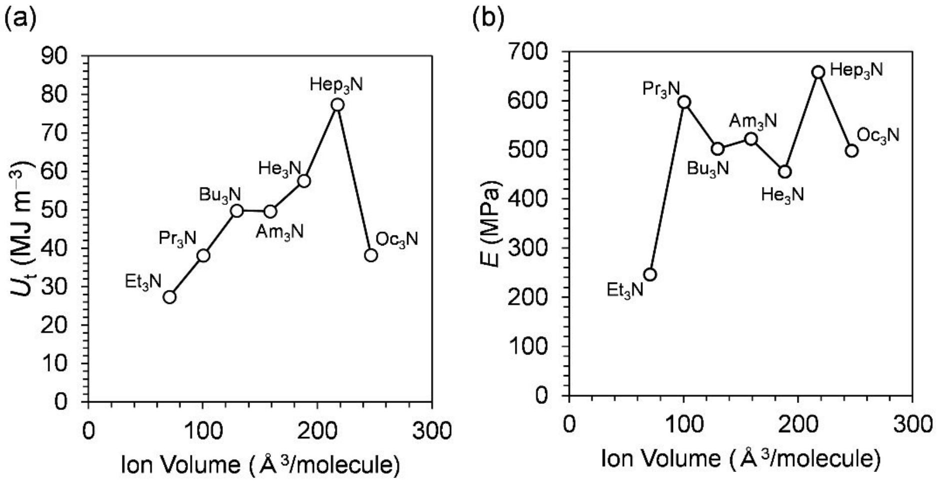

Figure S12. (a) The U_t and (b) E for pNBC-g-base as a function of ion volume.

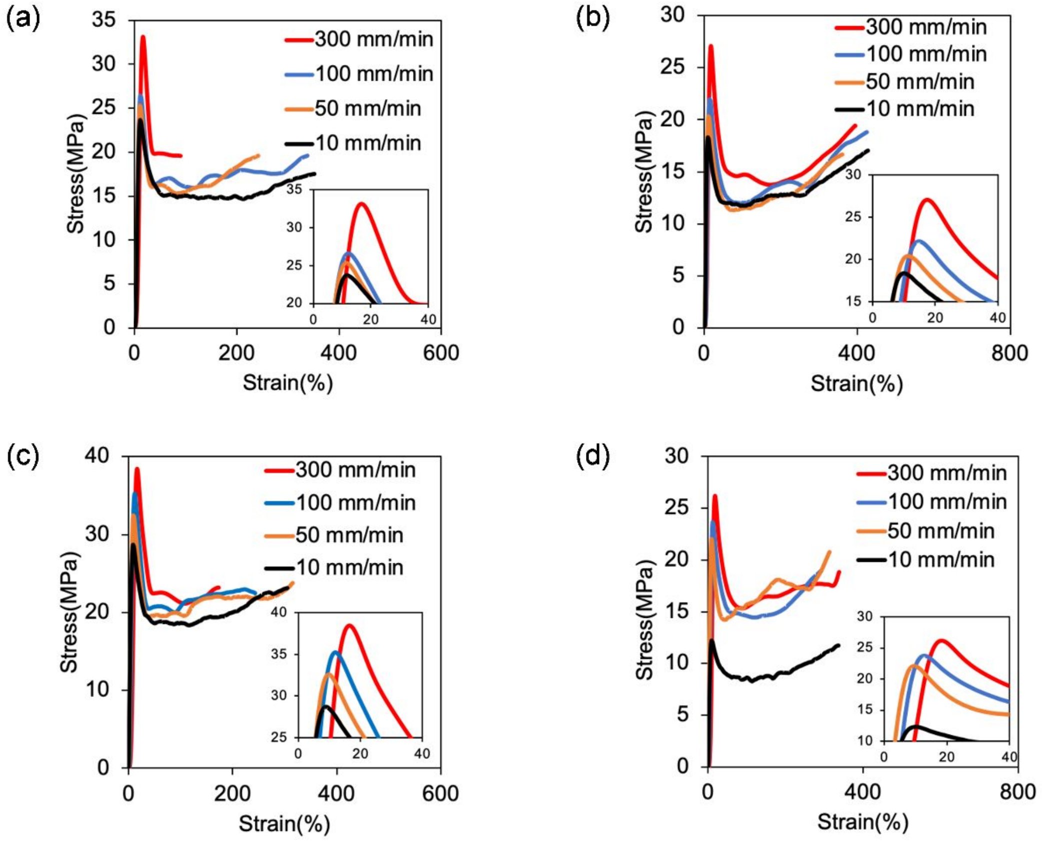

Figure S13. Stress-strain curves of (a) pNBC-g-Et3N, (b) pNBC-g-Hex3N, (c) pNBC-g-Hep3N, and (d) pNBC-g-Oc3N at different strain rate.

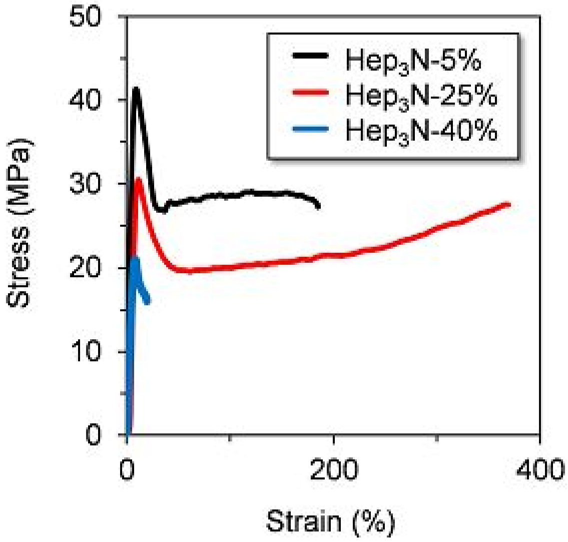

Figure S14. Stress-strain curves for pNBC-g-Hep$_3$N with neutralized by heptyl amine at base mole fractions.

4. Swelling study

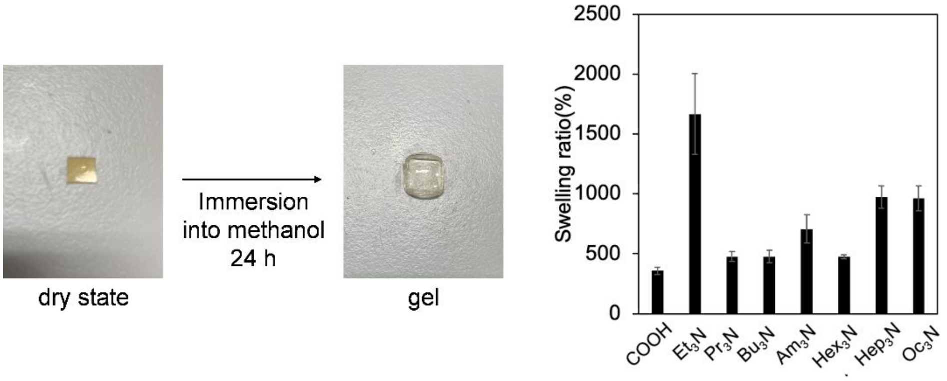

Figure S15. Swelling ratio for pNBC-g-base samples in methanol.

5. Rheology

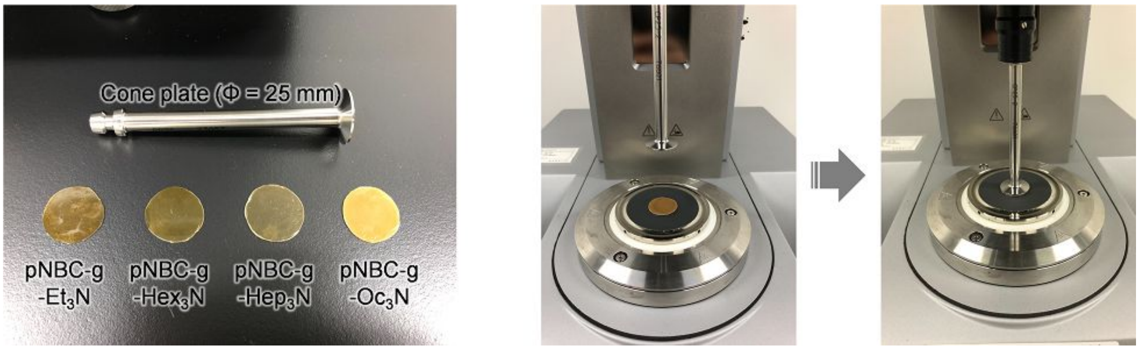

Figure S16. Photographs of a cone plate and round disc samples and sample loading for rheological measurements.

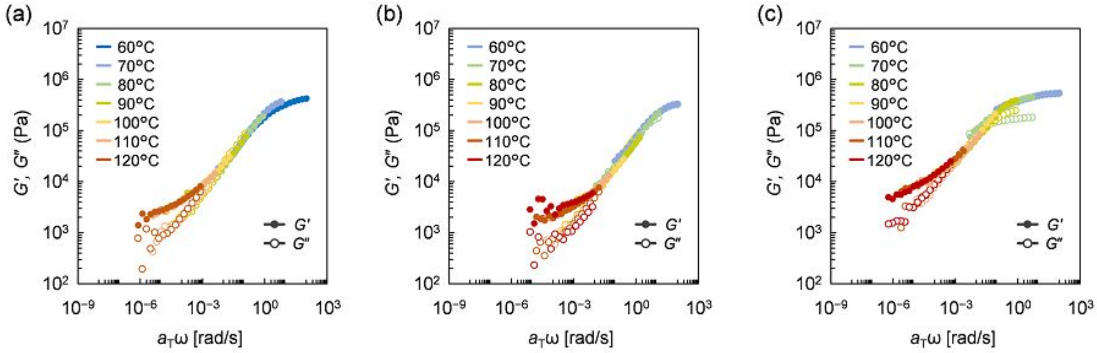

Figure S17. Frequency dependence of storage (G′) and loss modulus (G″) master curves at T_r = 60 °C for (a) pNBC-g-Et_3N, (a) pNBC-g-Hep_3N, and (c) pNBC-g-Oc_3N.

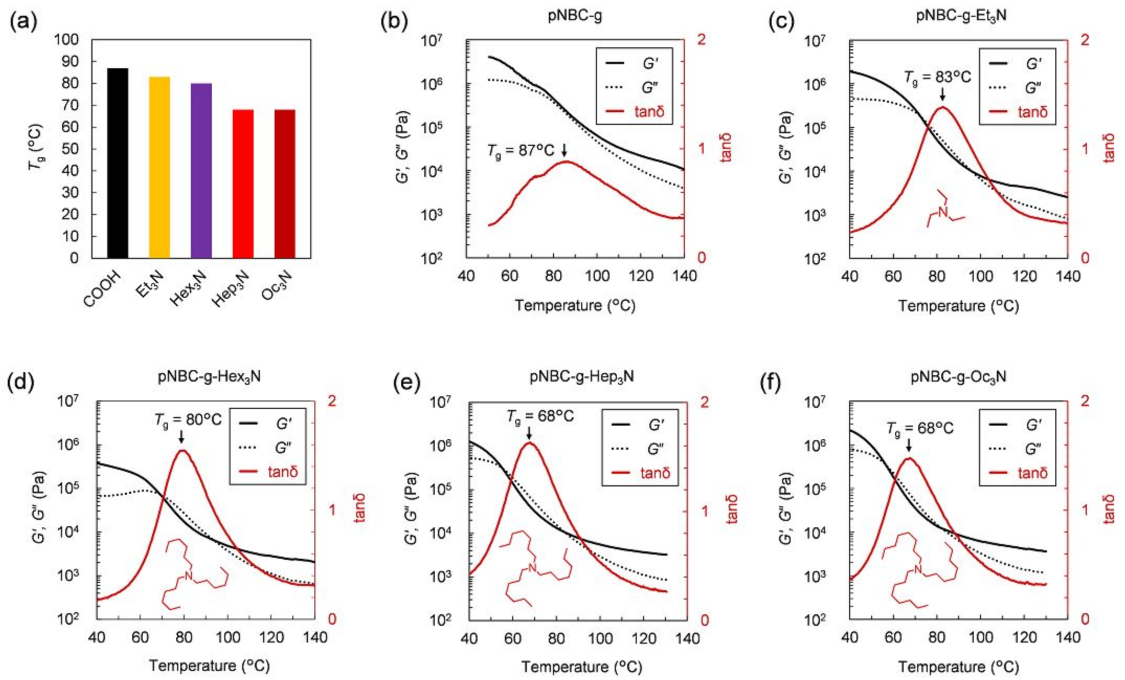

Figure S18. (a) Comparison of T_g values of pNBC-g-base with counterion sizes (Et_3N, Hex_3N, Hep_3N, Oc_3N). Temperature dependences of storage modulus (G′), loss modulus (G″), and tanδ for (b) pNBC-g, (c) pNBC-g-Et_3N, (d) pNBC-g-Hex_3N, (e) pNBC-g-Hep_3N, (f) pNBC-g-Oc_3N.
